# Uracil-tegafur and tamoxifen *vs* cyclophosphamide, methotrexate, fluorouracil, and tamoxifen in post-operative adjuvant therapy for stage I, II, or IIIA lymph node-positive breast cancer: a comparative study

**DOI:** 10.1038/sj.bjc.6605218

**Published:** 2009-07-28

**Authors:** Y Park, K Okamura, S Mitsuyama, T Saito, J Koh, S Kyono, K Higaki, M Ogita, T Asaga, H Inaji, H Komichi, N Kohno, K Yamazaki, F Tanaka, T Ito, H Nishikawa, A Osaki, H Koyama, T Suzuki

**Affiliations:** 1 Department of Surgery, Toho University School of Medicine, Sakura Hospital, 564-1 Shimoshizu, Sakura 285-8741, Japan; 2 Department of Surgery, Kumamoto Rosai Hospital, 1670 Takehara-machi, Yatsushiro 866-8533, Japan; 3 Department of Surgery, Kitakyushu Municipal Medical Center, 2-1-1 Bashaku, Kokurakita-ku, Kitakyushu 802-0077, Japan; 4 Department of Surgery, Omiya Red Cross Hospital, 8-3-33 Kamiochiai, Chuo-ku, Saitama 338-8553, Japan; 5 Department of Surgery, Saitama Social Insurance Hospital, 4-9-3 Kitaurawa, Urawa-ku, Saitama 330-0074, Japan; 6 Department of Surgery, Nippon Medical School, Chiba-hokuso Hospital, 1715 Kamagari, Inba-mura, Inba-gun, Chiba 270-1694, Japan; 7 Department of Surgery, Hiroshima Municipal Hiroshima Hospital, 7-33 Motoi-machi, Naka-ku, Hiroshima 730-8518, Japan; 8 Division of Mammary Endocrinology, Department of Surgery, National Sapporo Hospital, 4–2 Kikusui, Shiroishi-ku, Sapporo 003-0804, Japan; 9 Department of Breast and Thyroid Surgery, Kanagawa Cancer Center Hospital, 1-1-2 Nakao, Asahi-ku, Yokohama 241-0815, Japan; 10 Department of Surgery, Osaka Medical Center for Cancer and Cardiovascular Diseases, 1-3-3 Nakamichi, Higashinari-ku, Osaka 537-8511, Japan; 11 Department of Surgery, National Nara Hospital, 2-789 Shichijo, Nara 630-8053, Japan; 12 Department of Surgery, Hyogo Medical Center for Adult Disease, 13-70 Kitaoji-cho, Akashi 673-8558, Japan; 13 Department of Surgery, Asahikawa Medical College, 2-1-1-1 Midorigaoka-Higashi, Asahikawa 078-8510, Japan; 14 Department of Surgery, Fukui Red Cross Hospital, 2-4-1 Tukimi, Fukui 918-8501, Japan; 15 Department of Breast Surgery, Gifu Municipal Hospital, 7–1 Kashima-cho, Gifu 500-8513, Japan; 16 Department of Surgery, Hyogo Prefectural Amagasaki Hospital, 1-1-1 Higashidaimotsu-cho, Amagasaki 660-0828, Japan; 17 Department of Surgery, Research Institute for Radiation Biology and Medicine, Hiroshima University, 1-2-3 Kasumi, Minami-ku, Hiroshima 734-8553, Japan

**Keywords:** breast cancer, combination therapy, CMF, tamoxifen, randomised trial, uracil-tegafur

## Abstract

**Background:**

It has been reported that treatment with uracil-tegafur (UFT) has shown significantly better survival and relapse-free survival (RFS) than surgery alone. Therefore, we compared UFT with a combination therapy of cyclophosphamide, methotrexate, and fluorouracil (CMF) in patients who had undergone curative surgery for axillary lymph node-positive breast cancer.

**Methods:**

A total of 377 node-positive patients with stage I, II, or IIIA disease were registered from September 1996 through July 2000 and were randomly assigned to either 6 cycles of CMF or 2 years of UFT. In both arms, tamoxifen (TAM) was concurrently administered for 2 years. The primary end point in this study was the non-inferiority of UFT to CMF.

**Results:**

No statistically significant difference between the two groups was observed with regard to the 5-year RFS rate (72.2% in the UFT and 76.3% in the CMF). Adverse event profiles differed between the two groups, with a significantly lower incidence of leukopenia and anaemia in the UFT group, as well as anorexia, nausea/vomiting, stomatitis, and alopecia, which have implications for quality of life.

**Conclusion:**

UFT administered in combination with TAM holds promise in the treatment of lymph node-positive early breast cancer. On stratified analysis, the recurrence rate in the UFT group was found to be better in oestrogen receptor (ER)-positive patients. Tegafur-based treatment should be evaluated by a prospective randomised trial conducted in ER-positive patients.

The Early Breast Cancer Trialists’ Collaborative Group (EBCTCG) conducts a meta-analysis of randomised studies of post-operative adjuvant therapy for breast cancer every 5 years (EBCTCG, 2005). They reported that polychemotherapy (cyclophosphamide (CPA), methotrexate (MTX), fluorouracil (5-FU) ((CMF))-based; anthracycline-based; or other types of polychemotherapy) was associated with a decrease in the odds ratio of disease-free survival and an overall improvement in survival compared with no chemotherapy (EBCTCG, 1998).

The relatively recent addition of taxanes to anthracycline-based chemotherapy has markedly improved outcomes of post-operative chemotherapy for node-positive breast cancer (Ferguson *et al*, 2009). Furthermore, concomitant chemotherapy with trastuzumab, an anti-human epidermal growth factor receptor 2 (HER2) antibody, has been reported to substantially improve outcomes in resected HER2-positive breast cancer (Mackey *et al*, 2009). Selection of the most suitable drugs and regimens should be done in careful consideration of anticipated risks, expected benefits, and estimated costs.

Associated with fewer adverse events such as cardiotoxicity and bone marrow suppression compared with anthracycline- or taxane-based regimens, CMF therapy remains widely used as a therapeutic option. Furthermore, if therapeutic drugs could be administered orally instead of being intravenously infused, patients with breast cancer would benefit greatly because oral formulations are more convenient, are associated with fewer adverse events such as alopecia, and have higher compliance.

With regard to monotherapy regimens, oral fluoropyrimidines such as uracil-tegafur (UFT) (Taguchi, 1997), which are suitable for ambulatory-based treatment, have been used in Japan since the 1980s. Tashiro *et al* (1994) reported a response rate for UFT in patients with recurrent breast cancer of 39%, which is comparable with the 36% reported for CMF in patients with advanced or recurrent breast cancer in Japan (Nomura *et al*, 1994), as well as markedly lower toxicity for UFT than CMF. These results suggest that UFT likely prevents relapse as effectively as CMF in post-operative adjuvant therapy for breast cancer, but with a lower severity of adverse events.

Furthermore, in the adjuvant setting, treatment with UFT has shown significantly better survival and relapse-free survival (RFS) than surgery alone in prospective randomised clinical trials in breast cancer (Kasumi *et al*, 2003; Noguchi *et al*, 2005).

Here, we conducted a randomised study to compare UFT with CMF in patients with axillary lymph node-positive stage I, II, or IIIA breast cancer.

## Patients and methods

### Eligibility criteria

Eligibility criteria included (1) TNM stage I, II, or IIIA; (2) involvement of 1–9 axillary lymph nodes; (3) curative surgery; (4) age 20–65 years; (5) body weight ⩾40 kg; (6) normal haematological parameters as well as adequate hepatic and renal function; and (7) written informed consent. Patients with bilateral breast cancer, male breast cancer, inflammatory breast cancer, and double cancers were excluded, as were pregnant or lactating women with breast cancer. Patients were eligible regardless of their oestrogen receptor (ER) status. The cutoff level of ER was 13 fmol mg^−1^ soluble fraction in terms of enzyme immunoassay. Consecutive patients who met all of the eligibility criteria and received treatment as specified were included in the analysis.

### Study design

**Figure 1:**
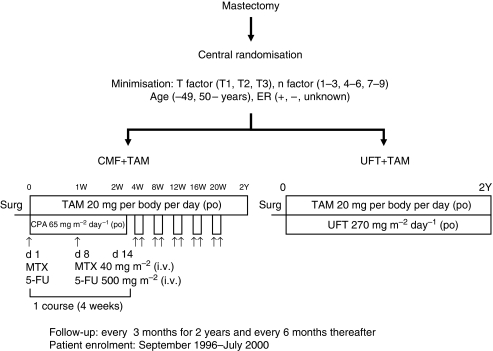
Study design. ER, oestrogen receptor; CMF, cyclophosphamide–methotrexate–fluorouracil; TAM, tamoxifen; UFT, uracil-tegafur; W, week; Y, year; Surg, surgery; po, per os; CPA, cyclophosphamide; d, day; MTX, methotrexate; 5-FU, fluorouracil; i.v., intravenous.

The study scheme is shown in Figure 1. The UFT (Taiho Pharmaceuticals Co. Ltd., Tokyo, Japan) group received tamoxifen (TAM, AstraZeneca K.K., Osaka, Japan) (20 mg day^−1^) in combination with UFT (270 mg m^−2^ day^−1^) for 2 years. The CMF group received TAM (20 mg day^−1^) for 2 years in combination with 6 cycles of CPA (Shionogi & Co. Ltd., Osaka, Japan) (65 mg m^−2^: days 1–14), MTX (Wyeth K.K., Tokyo, Japan) (40 mg m^−2^: days 1 and 8), and 5-FU (Kyowa Hakko Kirin Co. Ltd., Tokyo, Japan) (500 mg m^−2^: days 1 and 8), with the cycle repeated every 4 weeks.

Laboratory tests and observation of signs and symptoms were carried out every month in the UFT group for the first 6 months and thereafter at intervals of 3 months for the remainder of the 2-year treatment period, and on days 1, 8, and 15 of each cycle in the CMF group. Adverse events were assessed according to the grade classification of the Japan Society of Clinical Oncology, which are equivalent to the World Health Organization in all substantial aspects. Eligibility of subjects, cases of relapse, and deaths were clinically reviewed by the Data Review Committee. The study protocol was approved by the institutional review board of each participating institution. All procedures were carried out in accordance with the Declaration of Helsinki.

### Statistical considerations

The primary end point in this study was the non-inferiority of the UFT group to the CMF group, as measured by the RFS rate. The secondary end point was superiority with regard to a lower incidence of adverse events. Given that the EBCTCG study reported a 9.4±1.4% difference in 5-year recurrence-free survival rate in node-positive patients between the CMF therapy and control groups (EBCTCG, 1992), we adopted an approximate value of Δ10% to demonstrate clinical non-inferiority. We estimated a 5-year RFS rate of 70% in both the UFT and the CMF groups. As Δ10% corresponds to a hazard ratio (HR) of 1.43, demonstration of non-inferiority required the upper limit of the 90% confidence interval for the HR of the UFT group relative to the CMF group to be ⩽1.43. Demonstration of non-inferiority at a significance level of 0.05 (one-sided) with a power of 80% in turn required a sample size of 648 subjects. Therefore, we selected a sample size of 680 subjects, in consideration of the likely number of ineligible subjects and subjects not treated as specified.

Distribution of background factors and the incidence of adverse events were analysed using the *χ*^2^ test and Mann–Whitney *U*-test, respectively. Relapse-free survival time was defined as the period from the day of surgery to the day of final confirmation of a relapse-free status or to the first confirmation of relapse. For subjects who died of any cause other than relapse of the underlying disease, RFS time was defined as the period from the day of surgery to the day of death, with death considered as an event. Survival time was defined as the period from the day of surgery to the day of final confirmation of survival or to the confirmation of death from any cause.

Analyses in this study were performed on evaluable patients. Analyses were carried out using SAS software (version 6.12, SAS Institute, Cary, NC, USA), with the PHREG procedure used to estimate HR. In accordance with changes introduced by the ICH International Conference on Harmonization E9 (1998), the upper limit of the confidence intervals was changed to 95%. Both RFS and survival were estimated using the Kaplan–Meier method and analysed by the log-rank test. A *P*-value <0.05 was considered to indicate a statistically significant difference.

## Results

### Enrolment and patient characteristics

Patient characteristics are shown in Table 1. Recruitment began in September 1996 at 100 participating clinical sites in Japan. The recruitment period was extended in June 1988 from the originally planned 2 years to 4 years because the target number of subjects had not been reached. Despite this, only half the target sample size was achieved because of slow accrual and, as a result, recruitment was terminated in July 2000 with 377 enrolled patients. Among the 377 patients, 188 were randomised to the UFT group, with 3 excluded as being ineligible and 8 for protocol violations, leaving 177 patients for inclusion in the analysis. For the CMF group, 189 patients were randomised, with 7 excluded as being ineligible and 9 because of protocol violations, leaving 173 patients for analysis. No marked bias was observed in age distribution, hormone receptor status, number of involved axillary lymph nodes, or histological type. The frequency of ER-positive cases in the UFT and CMF groups was the same (51.4%).

### Efficacy

**Figure 2:**
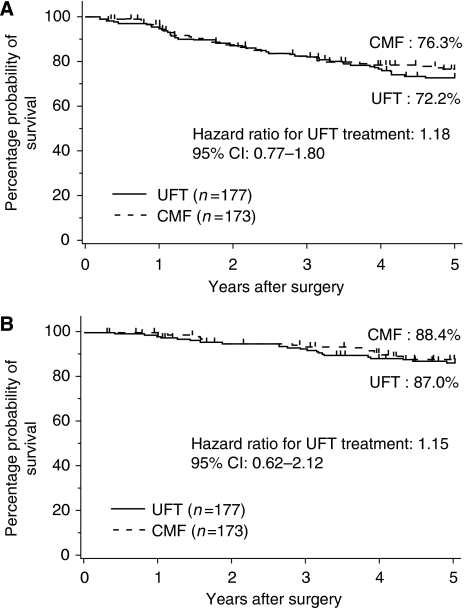
Relapse-free and overall survival. (**A**) Relapse-free survival; (**B**) overall survival. CMF, cyclophosphamide–methotrexate–fluorouracil; UFT, uracil-tegafur; CI, confidence interval.

**Figure 3:**
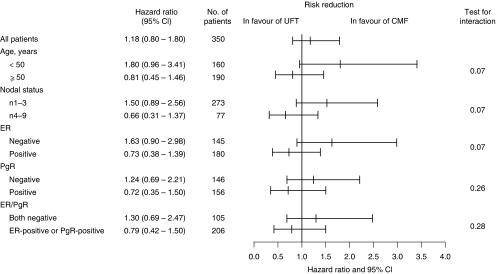
Relapse-free survival by age, number of involved nodes and ER, PgR, and ER/PgR status.

Both RFS and survival curves are shown in Figure 2. The 5-year RFS rates in the UFT and CMF groups were 72.2 and 76.3%, respectively, with no statistically significant difference between them (log-rank test, *P*=0.46). The HR for the UFT group relative to the CMF group in terms of RFS rate was 1.18 (95% CI: 0.77–1.80). Five-year survival rates in the UFT and CMF groups were 87.0 and 88.4%, respectively, showing no statistically significant difference between them, and the HR was 1.15 (log-rank test, *P*=0.66). Subset analysis showed that there were non-significant tendencies of interaction between RFS and age, number of involved lymph nodes and ER status (Figure 3).

The number of cases of relapse and the distribution of sites are shown in Table 2. There were 45 (25.4%) and 39 (22.5%) cases of relapse in the UFT and CMF groups, respectively. The most common initial site of relapse was soft tissue, followed by bone and visceral organs in both the groups. In addition, neither secondary breast cancer nor other malignancies were observed in both the groups. No difference in the number of cases or distribution of sites was observed between the two groups.

### Safety

Adverse events are shown in Table 3. Adverse events were reported in 88.1% (156 out of 177) of patients receiving UFT and in 98.8% (171 out of 173) of those receiving CMF, showing a significantly lower incidence in the UFT group (*P*<0.05). The incidence of leukopenia as well as haemoglobin, anorexia, nausea/vomiting, stomatitis, and alopecia was significantly higher in the CMF group, whereas that of liver dysfunction was significantly higher in the UFT group.

### Dose and concomitant medications

The mean total dose of UFT was 75.2% of the target dose, whereas the mean total doses of CPA, MTX, and 5-FU were 85.8, 83.3, and 83.7% of the target dose, respectively. The mean total dose of TAM in the UFT and CMF groups was 85.7 and 86.7% of the target dose, respectively, and thus was not different between the two groups.

Granulocyte colony-stimulating factors (G-CSFs) were used in 10 subjects (5.8%) in the CMF group, but not in any subjects in the UFT group. We used 5-hydroxytryptamine 3 (5-HT_3_) antagonists in 80 subjects (46.2%) in the CMF group, but in only 1 subject (0.6%) in the UFT group. The use of both G-CSFs and anti-emetics was statistically significantly less frequent in the UFT group (both *P*=0.001).

## Conclusion

In this study, we found no statistically significant difference in RFS among patients who had undergone curative surgery for axillary lymph node-positive breast cancer between those receiving UFT and CMF, at 72.2 *vs* 76.3%, respectively. In addition, the UFT group had significantly lower rates of adverse events compared with the CMF group (*P*<0.05). Moreover, G-CSFs were used by 0 and 5.8% of subjects who received UFT and CMF therapies, respectively, and 5-HT_3_ antagonists by 0.6 and 46.2%, respectively.

One particular limitation of this study warrants mention. Owing to its small sample size, this study did not have sufficient statistical power to demonstrate the non-inferiority of UFT to CMF. However, the HRs for RFS and survival calculated over 5 years were 1.18 and 1.15, respectively, which are consistent with the RFS and survival curves. Furthermore, the number of cases was sufficient enough to verify significant differences between the UFT and CMF groups with regard to adverse events. The UFT group had a significantly lower incidence of adverse events. These differences indicate the possibility of using UFT as a treatment option in place of CMF.

Similar findings were recently reported in a randomised study of stage I–IIIA node-negative, pathologically high-risk breast cancer, which compared a 2-year administration of UFT with 6 cycles of CMF (Watanabe *et al*, 2009). Results indicated that the efficacy of UFT as an adjuvant treatment was comparable with that of CMF. On the other hand, Cancer and Leukemia Group B (CALGB) reported that a 4.5-month administration of capecitabine was inferior to CMF/AC (doxorubicin (ADM)+CPA) in elderly breast cancer patients (Muss *et al*, 2009). This disagreement among results, including those of this study, might be partly explained by the difference in the duration of treatment with oral fluoropyrimidines.

An additional finding of our study was that subset analysis indicated a tendency of interaction among age, number of involved lymph nodes, or ER status and RFS, suggesting that these subsets may affect therapeutic effect. Among these three subsets, it is unclear why UFT was superior to CMF in patients with four or more metastatic axillary lymph nodes. With regard to age in pre-menopausal women, relapse prevention by CMF is mainly attributable to its chemical oophorectomy effect, but in post-menopausal women primarily to other mechanisms, such as cytotoxic effects.

In contrast, UFT is reported to have anti-angiogenic effects in addition to its cytotoxic effects based on the evaluation in BALB/c mouse models (Yonekura *et al*, 1999; Munoz *et al*, 2006). These findings may explain the higher efficacy of UFT than CMF in patients aged ⩾50 years. With regard to ER status, our findings support previous findings that concurrent treatment with UFT and TAM yields better outcomes in patients with ER-positive breast cancer than does monotherapy with TAM (Noguchi *et al*, 2005). Given the finding by Albain (1989) that the CAF–TAM combination treatment is more effective in sequential than in concurrent administration, combination treatment with anthracycline-based infusional therapy with TAM is believed to have equivalent or lower efficacy when administered concurrently than sequentially. However, the validity of the concurrent approach *in vitro* has been demonstrated (Kurebayashi *et al*, 2007). The combination of 4-hydroxy-TAM (4OHT) and 5-FU had an additive effect in inhibiting the proliferation of ER-*α*-positive cells, whereas that of 4OHT and ADM worked antagonistically. With regard to changes in gene expression for susceptibility or tolerance to TAM, 5-FU, or ADM, 5-FU did not change the expression level of TAM-related genes, whereas 4OHT significantly inhibited thymidylase synthase, which is a key enzyme in the anti-tumour activity of 5-FU, and thereby enhanced the anti-tumour effect of 5-FU. In contrast, ADM did not change gene expression for TAM susceptibility, but did increase the expression of some genes for TAM tolerance. The authors considered this to indicate the fact that ADM in combination with 4OHT may exert a rather negative anti-tumour effect. Furthermore, anthracyclines are reportedly less effective against HER2-negative than HER2-positive breast cancers (Gennari *et al*, 2008), whereas the relapse-preventative effect of UFT is not affected by HER2 status (Toi *et al*, 2007).

The Consensus Guideline (Goldhirsch *et al*, 2007) established at the St Gallen Conference states that no therapeutic modality has been established for HER2-negative and hormone receptor-positive intermediate-risk breast cancer. The question of whether post-operative endocrine therapy alone is sufficient in these patients or should be supplemented with chemotherapy remains unanswered. As mentioned above, as UFT can prevent ER-positive breast cancer from recurring when combined with hormone therapy, regardless of HER2 status, it may provide a useful therapeutic option for patients with intermediate-risk breast cancer.

In conclusion, these findings suggest that UFT may hold promise as a treatment for the prevention of relapse of node-positive early breast cancer. Tegafur-based treatment should be evaluated by a prospective randomised trial conducted in ER-positive patients.

## Figures and Tables

**Table 1:** Patient characteristics

	**CMF**	**UFT**
No. of enrolled patients	189	188
No. of eligible patients	182	185
No. of evaluable patients	173	177
		
*Age (mean±s.d.)*	51.0±7.7	51.7±7.8
<50 years	88	88
⩾50 years	85	89
		
Body surface area (m^2^, mean±s.d.)	1.47±0.11	1.48±0.11
		
*T*		
T1	36	31
T2	122	132
T3	15	14
		
*n*		
1–3	135	138
4–6	26	25
7–9	12	14
		
*Hormone receptor status*		
ER(−) and PgR(−)	52	53
Others	121	124
		
ER(−)	73	72
ER(+)	89	91
Unknown	11	14
		
PgR(−)	73	73
PgR(+)	78	78
Unknown	8	6
		
*Histological pattern*		
Invasive ductal ca.	165	160
Lobular ca.	5	7
Others	3	10

ca.=carcinoma; CMF=cyclophosphamide–methotrexate–fluorouracil; ER=oestrogen receptor; PgR=progesterone receptor; UFT=uracil-tegafur.

**Table 2:** Sites of relapse

	**CMF**	**UFT**
No. of evaluable patients	173	177
No. of relapse cases	39 (22.5%)	45 (25.4%)
*Soft tissue*	18 (10.4%)	23 (13.0%)
Skin and subcutaneous tissues	9 (5.2%)	12 (6.8%)
Lymph node	10 (5.8%)	12 (6.8%)
Contralateral breast	1 (0.6%)	
Bone	15 (8.7%)	12 (6.8%)
*Visceral organs*	14 (8.1%)	19 (10.7%)
Lung and pleura	8 (4.6%)	12 (6.8%)
Liver	7 (4.0%)	7 (4.0%)
Brain	2 (1.2%)	2 (1.1%)
Others	1 (0.6%)	

CMF=cyclophosphamide–methotrexate–fluorouracil; UFT=uracil-tegafur.

**Table 3:** Adverse events

	**CMF (*n*=173)**						**UFT (*n*=177)**						
	**Grade**						**Grade**						
	**Unknown**	**1**	**2**	**3**	**4**	**Incidence (%)**	**Unknown**	**1**	**2**	**3**	**4**	**Incidence (%)**	***P-*value^a^**
Leukopenia		87	50	6		82.7		49	10			33.3	*P*<0.001
Thrombocytopenia		21	2			13.3		35	2			20.9	
Haemoglobin		52	2	1		31.8		11	2			7.3	*P*<0.001
AST		45	11			32.4		45	16		1	35.0	
ALT		47	16			36.4		51	16	2		39.0	
Hepaplastin decrease		1				0.6		9				5.1	*P*<0.05
Total bilirubin		3				1.7		43	3	1		26.6	*P*<0.001
ALP		4				2.3		25				14.1	*P*<0.001
BUN		2	1			1.7		10				5.6	
Creatinine						—		1				0.6	
Anorexia		62	18	6		49.7	1	34	4	1		22.6	*P*<0.001
Nausea/vomiting		84	28	8		69.4	1	32	6	1		22.6	*P*<0.001
Diarrhoea	1	8				5.2	2	11				7.3	
Stomatitis		17	3			11.6		2	3			2.8	*P*=0.002
Pigmentation		7	2			5.2		12	4			9.0	
Numbness in fingers		1				0.6			1			0.6	
Alopecia		57	11			9.3		4				2.3	*P*<0.001

a *χ*^2^ test.

ALP=alkaline phosphatase; ALT=alanine aminotransferase; AST=aspartate aminotransferase; BUN=blood urea nitrogen; CMF=cyclophosphamide–methotrexate–fluorouracil; UFT=uracil-tegafur.

**Table A1:** 

Asahikawa Medical College	Toho University School of Medicine, Sakura Hospital
Kushiro Rosai Hospital	Nippon Medical School, Chiba-hokuso Hospital
National Sapporo Hospital	Cancer Research Institute Hospital
Asahikawa Municipal Hospital	School of Medicine, Keio University
Sapporo Municipal Hospital	Tokyo Medical University
Nikko Memorial Hospital	Tokyo Women's Medical University School of Medicine
Aomori Prefectural Central Hospital	Tokyo Women's Medical University Daini Hospital
Akita University School of Medicine	Toranomon Hospital
Ota Nishinouchi Hospital	Japan Red Cross Hospital Medical Center
National Sendai Hospital	Mitsui Memorial Hospital
Sendai Municipal Hospital	Odawara Municipal Hospital
Fukushima Medical University	Kanagawa Cancer Center Hospital
Hoshi General Hospital	National Sagamihara Hospital
Yamagata University School of Medicine	Showa University Fujigaoka Hospital
Omiya Red Cross Hospital	Koseiren Takaoka Hospital
Ojiya General Hospital	Fukui Red Cross Hospital
Kasukabe Municipal Hospital	National Kanazawa Hospital
Gunma University School of Medicine	Yaizu Municipal Hospital
Jichi Medical School	Aichi Cancer Center Hospital
Saitama Social Insurance Hospital	Kouseiren Kosei Hospital
Tochigi Cancer Center Hospital	Iida Municipal Hospital
Saiseikai Utsunomiya Hospital	Gifu Municipal Hospital
Kameda General Hospital	Koseiren Gihoku Hospital
Chiba Cancer Center Hospital	Yokkaichi Municipal Hospital
The Jikei University Kashiwa Hospital	Shinshu University School of Medicine
Medical School, Nagoya City Hospital	Nagano Red Cross Hospital
Nagoya First Red Cross Hospital	Omoto Hospital
Nagoya University School of Medicine	Kawasaki Medical School
Yamada Red Cross Hospital	Hiroshima Prefectural Hiroshima Hospital
Kyoto Municipal Hospital	National Iwakuni Hospital
Kyoto University Faculty of Medicine	National Kure Medical Center
Kyoto Prefectural University of Medicine	Hiroshima Municipal Hiroshima Hospital
Shiga Adult Disease Center	Hiroshima Municipal Asa Hospital
Osaka Medical College	Research Institute for Radiation and Medicine, Hiroshima University
Osaka Police Hospital	Anan Central Hospital for the Medical Association
Osaka City University Medical School	Kochi Medical School
Osaka University School of Medicine	National Shikoku Cancer Center
Osaka Medical Center for Cancer and Cardiovascular Diseases	Matsuyama Red Cross Hospital
Kansai Medical University	School of Medicine, The University of Tokushima
Kinki University School of Medicine	Oita Prefectural Hospital
National Osaka Medical Center	Kitakyushu Municipal Medical Center
National Nara Hospital	Kyushu University Faculty of Medicine
Sakai Municipal Hospital	National Kyushu Medical Center
Toyonaka Municipal Hospital	National Miyakonojo Hospital
Yao Municipal Hospital	Saga Prefectural Koseikan Hospital
Suita Municipal Hospital	Ngasaki Municipal Hospital
Kansai Rosai Hospital	Nagasaki Red Cross Hospital
Hyogo Prefectural Amagasaki Hospital	School of Medicine Fukuoka University
Kobe Municipal Central Hospital	Kumamoto Rosai Hospital
Kinki Central Hospital	
Hyogo Medical Center for Adult Disease	

## Appendix

### Participating institutions

Table A1
