# Uracil-tegafur and tamoxifen versus cyclophosphamide, methotrexate, fluorouracil, and tamoxifen in postoperative adjuvant therapy for stage I, II, or IIIA lymph node-positive breast cancer: a comparative study

**DOI:** 10.1038/sj.bjc.6605295

**Published:** 2009-09-08

**Authors:** Y Park, K Okamura, S Mitsuyama, T Saito, J Koh, S Kyono, K Higaki, M Ogita, T Asaga, H Inaji, H Komichi, N Kohno, K Yamazaki, F Tanaka, T Ito, H Nishikawa, A Osaki, H Koyama, T Suzuki

C**orrection to**: *British Journal of Cancer* (2009) **101**, 598–604; doi:10.1038/sj.bjc.6605218 www.bjcancer.com

Upon publication of this paper in Volume 101, the authors noticed an error in the data contained within Table 3, taken from their original manuscript. The incidence of alopecia in CMF group should have been 39.3%, not 9.3%. The correct data are now shown, below.

## Figures and Tables

**Table 3:** Adverse events

	**CMF (*n*=173)**						**UFT (*n*=177)**						
	**Grade**						**Grade**						
	**Unknown**	**1**	**2**	**3**	**4**	**Incidence (%)**	**Unknown**	**1**	**2**	**3**	**4**	**Incidence (%)**	***P-*value^a^**
Leukopenia		87	50	6		82.7		49	10			33.3	*P*<0.001
Thrombocytopenia		21	2			13.3		35	2			20.9	
Haemoglobin		52	2	1		31.8		11	2			7.3	*P*<0.001
AST		45	11			32.4		45	16		1	35.0	
ALT		47	16			36.4		51	16	2		39.0	
Hepaplastin decrease		1				0.6		9				5.1	*P*<0.05
Total bilirubin		3				1.7		43	3	1		26.6	*P*<0.001
ALP		4				2.3		25				14.1	*P*<0.001
BUN		2	1			1.7		10				5.6	
Creatinine						—		1				0.6	
Anorexia		62	18	6		49.7	1	34	4	1		22.6	*P*<0.001
Nausea/vomiting		84	28	8		69.4	1	32	6	1		22.6	*P*<0.001
Diarrhoea	1	8				5.2	2	11				7.3	
Stomatitis		17	3			11.6		2	3			2.8	*P*=0.002
Pigmentation		7	2			5.2		12	4			9.0	
Numbness in fingers		1				0.6			1			0.6	
Alopecia		57	11			39.3		4				2.3	*P*<0.001

Abbreviations: ALP=alkaline phosphatase; ALT=alanine aminotransferase; AST=aspartate aminotransferase; BUN=blood urea nitrogen; CMF=cyclophosphamide–methotrexate–fluorouracil; UFT=uracil-tegafur.

a *χ*^2^ test.

